# Current status and clinical applications of tissue engineering of the gastrointestinal tract: a systematized narrative review

**DOI:** 10.3389/fgstr.2023.1277094

**Published:** 2023-11-07

**Authors:** Yilin Liu, Lynn Chong, Matthew Read

**Affiliations:** ^1^ Melbourne Medical School, Faculty of Medicine, Dentistry and Health Sciences, The University of Melbourne, Melbourne, VIC, Australia; ^2^ Department of Surgery, St Vincent’s Hospital, University of Melbourne, Fitzroy, VIC, Australia; ^3^ Department of Hepatobiliary and Upper Gastrointestinal Surgery, St Vincent’s Hospital, Fitzroy, VIC, Australia

**Keywords:** tissue engineering, regenerative medicine, gastrointestinal tract, stem cells, organoids, scaffolds

## Abstract

**Background:**

Since the advent of regenerative medicine, tissue engineering of the gastrointestinal tract (GIT) has been extensively studied in laboratory animals and humans. Various biologic scaffolds and cell sources have been trialed to repair or reconstruct different GIT defects. Achievements in this field have led to novel approaches in curing GIT diseases and circumventing the morbidity-related complications associated with current therapy.

**Objective:**

This review aims to describe recent advances in GIT tissue engineering, with an emphasis on technologies with potential for clinical use.

**Methods:**

A literature search was conducted in Ovid MEDLINE^®^ ALL for relevant studies (2000–September 2023) using the keywords “tissue-engineering”, “scaffolds”, “organoids”, “cell-therapy”, “esophagus”, “stomach”, “small intestine”, “colon”, “rectum”, and “anus”. Articles were included if they were *in vivo* animal studies or clinical studies written in English that investigated tissue engineering for treating GIT defects.

**Results:**

A total of 836 articles were identified in the initial search. Following duplicate removal, abstract, and full-text screening, 48 articles were included in the final review. Many studies on esophageal defects thus far have described the success of covering partial-thickness defects with autologous cell sheets and closing full-thickness defects with decellularized scaffolds in both animals and humans. A limited number of reports have also demonstrated the *de novo* organogenesis of the esophagus to repair short-segment circumferential esophageal defects with autologous pluripotent cells and scaffolds. In the stomach, multiple animal studies have reported on the feasibility of gastric epithelium regeneration using multipotent cells and/or scaffolds to correct partial- and full-thickness defects. One study observed the regeneration of whole-layer stomach defects using the organoids-on-polymer approach. Similarly, in the intestine, pluripotent cells and scaffolds were shown to effectively repair both partial- and full-thickness defects. Animal experiments have produced tissue-engineered small intestines (TESI) with the organoids-on-polymer approach. Furthermore, in the rectum and anus, mesenchymal stem cell therapies with or without bioscaffolds have shown promise for treating full-thickness defects, as demonstrated in multiple human trials.

**Conclusion:**

Tissue-engineering approaches for repairing various types of GI defects in the esophagus, stomach, intestines, rectum, and anus have been extensively explored in animal models, with promising outcomes. Moreover, successful human trials have demonstrated the feasibility of reconstructing esophageal, rectal, and anal defects using these innovative approaches. Technologies such as mesenchymal stem cells, decellularization, organoids, and cell sheets are the most promising and closer to clinical translation. Collaboration between gastrointestinal surgery and regenerative medicine is expected to bring about novel therapeutic modalities in the future.

## Highlights

- Gastrointestinal tissue engineering has the potential to offer novel therapeutic solutions to repair various gastrointestinal defects and avoid the morbidity-related complications associated with current therapy.
- Many studies on esophageal defects have described the success of repairing partial- and full-thickness defects with autologous cell sheets and decellularized scaffolds in both animals and humans.
- A limited number of reports have also demonstrated the *de novo* organogenesis of the esophagus with autologous pluripotent cells and scaffolds to repair long-segment circumferential defects.
- In the stomach, multiple animal studies reported the feasibility of repairing partial- and full-thickness defects with multipotent cells and/or scaffolds. One study observed the regeneration of whole-layer stomach defects using the organoids-on-polymer approach.
- In the intestine, pluripotent cells and scaffolds were similarly used to successfully repair partial- and full-thickness defects. Animal experiments have produced tissue-engineered small intestines with the organoids-on-polymer approach.
- In the rectum and anus, mesenchymal stem cell therapies with or without bioscaffolds have shown promise for treating full-thickness defects (e.g., perianal fistulas), as demonstrated in multiple human trials.
- Collaboration between gastrointestinal surgery and regenerative medicine is fundamental in bringing novel therapeutic modalities in the future.

## Introduction

The gastrointestinal tract (GIT) is a non-redundant organ with a limited ability to regenerate. The partial or complete loss of any gastrointestinal (GI) segment has devastating and potentially life-threatening sequelae. Partial-thickness defects, such as post-endoscopic submucosal dissection (ESD), can lead to fibrosis and refractory strictures (1). Full-thickness defects, such as perforations, leaks, and fistulas, can occur due to various disease processes, trauma, or certain medical procedures, causing significant morbidity and potential mortality (2, 3). In addition, whole circumferential defects following surgical resection of the diseased GI segment are often associated with significant complications, leading to poor quality of life (4). The current medical and surgical treatments are suboptimal and are associated with various complications. There are unmet clinical needs that necessitate the development of alternative approaches for managing GI defects.

Tissue engineering and regenerative medicine are rapidly evolving fields that combine cell biology, materials science, and physiology to develop functional substitutes that either enhance repair in damaged sites or create constructs to replace the deficient tissue. Although the specific methods and materials used in tissue engineering can vary, all involve three primary elements: the cell, the supporting scaffolds, and the environmental modulator, the last of which serves to integrate and regulate the functional behavior of the first two (4).

In recent years, achievements have been made in the field of gut bioengineering that hold great promise for the development of therapeutic solutions to various GI defects and avoiding complications associated with current therapy. The purpose of this review is to describe the recent advances in tissue engineering of the gastrointestinal tract, with an emphasis on technologies that are closer to clinical translation. Some key factors to consider and challenges to overcome when transitioning GIT tissue engineering toward clinical translation will also be explored.

## Methods

A narrative review of studies on GIT tissue engineering that took a systematic approach and was in accordance with PRISMA guidelines was conducted. A literature search (**Appendix 1**) was conducted in September 2023 using Ovid MEDLINE^®^ ALL. Keywords included “tissue-engineering”, “regenerative medicine”, “scaffolds”, “organoids”, “cell- and tissue-based therapy”, “esophagus”, “stomach”, “small intestine”, “colon”, “rectum”, and “anus”. The search results were screened for inclusion and exclusion criteria (**Table 1**) and assessed for relevance by a single reviewer (YL). Studies were considered relevant if they were *in vivo* animal studies or clinical studies that investigated tissue engineering for treating GI defects.

**Table 1:** Inclusion and exclusion criteria for study selection.

Inclusion criteria	Exclusion criteria
• *In vivo* animal studies or clinical trial • Studies on gastrointestinal defects (partial-thickness defects, full-thickness patch defects, or whole-layer circumferential defects) • Studies on tissue engineering techniques to repair GI defects • Written in the English language • Published after 2000 • Full-text article	• *In vitro*-only experiments • Studies that were not primary research, e.g., reviews, letters, and editorials • Studies that did not assess the reconstructed gastrointestinal wall properties • Cell culture, organoids, or scaffolds manufacturing techniques

## Results

**Figure 1:**
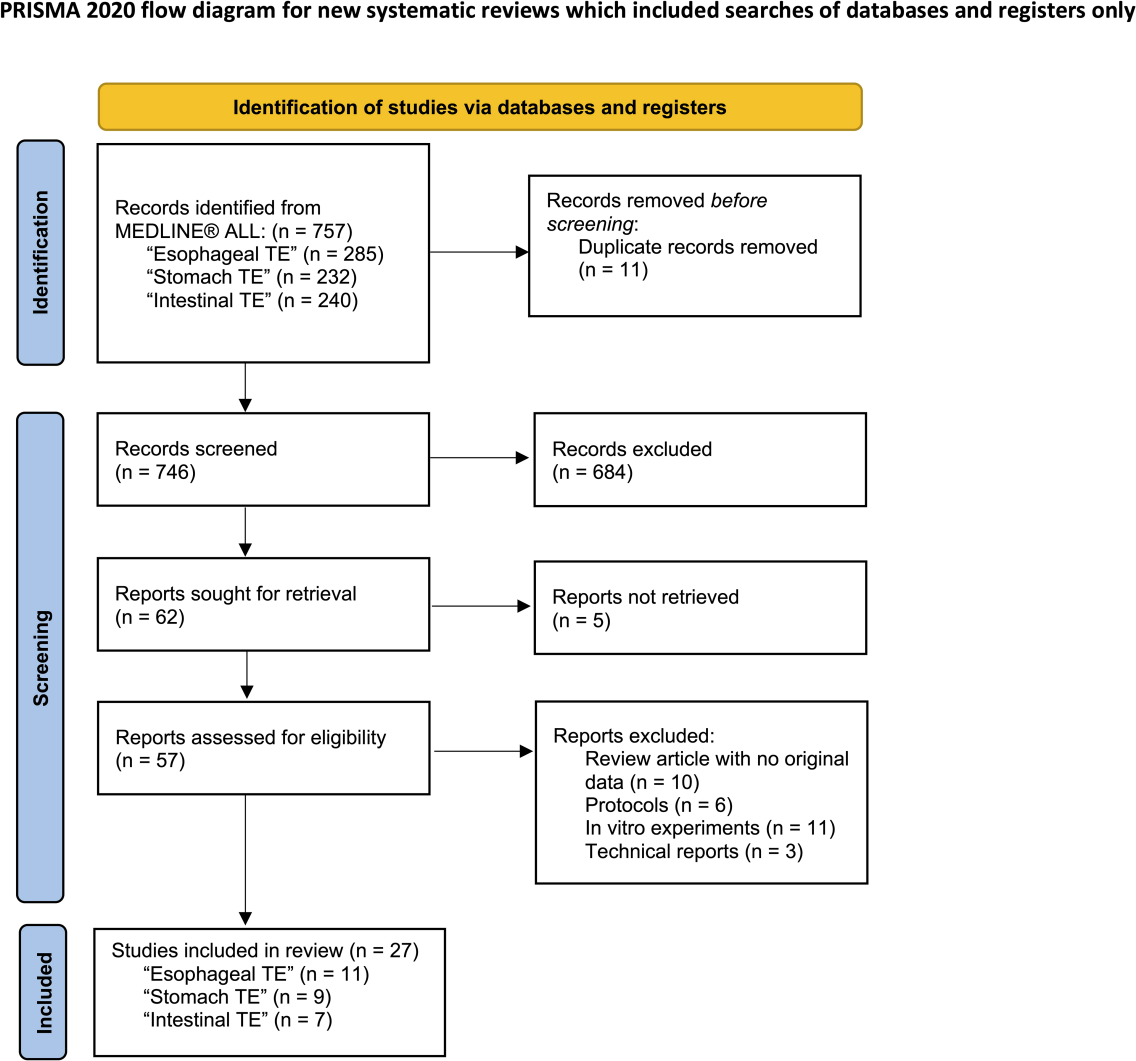
PRISMA flow diagram presenting the review process. TE, Tissue engineering.

The review process is summarized in the PRISMA flow diagram in **Figure 1**. A total of 288 articles on the esophagus, 233 articles on the stomach, 247 articles on the intestines, and 68 articles on the rectum and anus were initially identified through database searching. Duplications were then removed in EndNote (version X9.3.3). After screening by title and abstract, 57 full-text articles were retrieved and further assessed using the eligibility criteria outlined in **Table 1**. The reference lists of papers were also reviewed for additional relevant publications. Ultimately, 14 articles on the esophagus, 10 articles on the stomach, 7 articles on the intestines, and 17 articles on the rectum and anus were included in the final review (**Figure 1**). The first author, publication year, animal species or patient details, defect types, cell source, scaffolds, methodology, and outcome were collected from all included papers. Studies were grouped according to the anatomical locations and the type of defects investigated (**Tables 2**–**6**).

**Table 2:** Summary of *in vivo* animal studies on esophageal tissue engineering.

Author (year)	Cell source	Scaffold	Animal model	Methodology	Outcome	Limitations
Partial-thickness defects						
Ohki et al. (2006) (1)	Autologous oral mucosal epithelial cell sheets	–	Canine	Transplant tissue-engineered cell sheet endoscopically post semi-circumferential ESD	Intact stratified squamous cells and complete healing by 4 weeks	Very short survival follow-up time
Honda et al. (2010) (5)	Adipose tissue-derived stromal cells (ADSCs)	–	Canine	Inject ADSCs endoscopically to submucosa after circumferential esophageal ESD	Significantly reduced luminal narrowing in the study group	Animal study with small sample size (*n *= 5)
Kanai et al. (2012) (6)	Autologous epidermal cell sheets	–	Porcine	Transplant cell sheet endoscopically to the lesion after circumferential esophageal ESD	The occurrence of post-ESD strictures was significantly lower in the cell-sheet group	Animal study with small sample size (*n *= 8)
Nieponice et al. (2009) (7)	–	Dermal ECM	Canine	Place ECM scaffolds endoscopically after circumferential esophageal EMR	ECM scaffolds prevented clinically significant esophageal stenosis	Difficult to secure the ECM scaffold at the EMR site
Full-thickness defects						
Badylak et al. (2000) (8)	–	Porcine-derived ECM	Canine	Repair 5 cm full-thickness patch defects (> 40% esophageal circumference) with ECM scaffolds	Complete squamous epithelium and five-layer esophageal wall after 50 days	ECMs are limited for partially circumferential repair
Long-segment circumferential defects						
Catry et al. (2017) (9)	Autologous MSCs	Porcine-derived ECM	Porcine	MSC-seeded matrices or matrices alone were used to repair 3 cm-long circumferential esophageal defects in mini pigs	Mature squamous epithelium and desmin-positive cells were observed only in the MSC-seeded matrix group	Limited follow-up time
La Francesca et al. (2018) (10)	Autologous adipose-derived mesenchymal stem cells (ADMSCs)	Synthetic polyurethane electrospun grafts	Porcine	Electrospun polyurethane conduits seeded with aMSCs were placed in pigs underwent 4 cm–4.5 cm circumferential esophageal resections	Squamous esophageal mucosa, submucosa, and smooth muscle layers formed at 2.5 months	Definitive histologic data is limited at various time points
Levenson et al. (2022) (11)	Autologous bone marrow MSCs	Allogenic decellularized esophagus	Porcine	A total of 18 pigs underwent 5 cm circumferential esophageal resections and divided into four experimental groups according to mesenchymal stromal cells recellularization and omental maturation	At 6 months, the graft area showed a tissue-specific regeneration with a mature epithelium and muscular cells; but no additional benefit was observed in terms of omental maturation and MSC recellularization	A total of 13 out of 18 pigs experienced graft stenosis following stent migration, suggesting the difficulty of holding the graft in place
Sundaram et al. (2022) (12)	Autologous adipose-derived mesenchymal stem cells (ADMSCs)	Polyurethane tubular mesh cell delivery device (Cellframe™ Technology)	Porcine	A total of 12 mini pigs underwent 5 cm esophageal resections and were implanted with a Cellspan Esophageal Implant™ (CEI)	Esophageal tissue regenerated and continued to remodel over the course of 1-year survival with full layers regrown	High proportion of stent migration due to the use of human stents in pigs

"-" means not applicable.

**Table 3:** Summary of human studies on esophageal tissue engineering.

Author (year)	Cell source	Scaffold	Patient number	Methodology	Outcome	Limitations
Partial-thickness defects						
Ohki et al. (2012) (13)	Autologous oral mucosal epithelial cell sheets	–	9	Transplant tissue- engineered cell sheet endoscopically post semi-circumferential ESD	Complete re-epithelization within 3.5 weeks without esophageal strictures, dysphagia or any serious complications	Limited practical utility due to the expertise required for cell sheet manufacturing
Yamaguchi et al. (2017) (14)	Autologous oral mucosal epithelial cell sheets	–	10	Transplant tissue- engineered cell sheet endoscopically post complete circular or semicircular ESD	Post-ESD ulcer healing at 36 days without significant complications and 60% without stenosis; cell sheet preparation at distant sites and transportation by air feasible	Factors associated with esophageal stricture despite cell sheet transplantation remain to be determined
Full-thickness defects						
Nieponice et al. (2014) (2)	–	Porcine urinary bladder ECM	4	Suture ECM patches to large, full-thickness esophageal defects	Favorable clinical outcome in all cases with complete mucosal remodeling and epithelialization at 2 months post ESD	Small patient sample (*n *= 4) and heterogeneity of the clinical cases
Long-segment circumferential defects						
Dua et al. (2016) (4)	Autologous platelet rich plasma	AlloDerm™	1	Transplant AlloDerm™ with autologous platelet-rich plasma to a patient with a 5 cm circumferential defect	Full-thickness esophagus regenerated at 3.5 years with normal five-layer wall and peristaltic motility	Single case report, labor intensive, prolonged period of stenting
Aho et al. (2021) (15)	Autologous adipose-derived mesenchymal stromal cells (ADMSCs)	Polyurethane tubular mesh cell delivery device (Cellframe™ Technology)	1	Transplant Cellspan esophageal implant (CEI) to a patient with a 4 cm circumferential defect	Complete luminal epithelialization and partial esophageal tissue regeneration at 7.5 months	Single case report with a short follow-up period as the patient passed away because of a stroke

"-" means not applicable.

**Table 4:** Summary of *in vivo* animal studies on stomach tissue engineering.

Author (year)	Cell source	Scaffold	Animal model	Methodology	Outcome	Limitations
Partial-thickness defects						
Asakarov et al. (2008) (16)	Multipotent mesenchyme stromal cells (MMSCs)	–	Rats	Regeneration of experimental indolent stomach ulcer with MMSCs	MMSCs promote gastric mucosa healing with full epithelialization observed on day 30	Very short survival follow-up time
Nishida et al. (2008) (17)	Bone marrow cells and gastric myofibroblasts	–	Mice	Inject BM-derived cells, myofibroblasts, or saline around acetic acid-induced gastric ulcers	Ulcer healing was significantly promoted by the injection of BM-derived cells	Multipotentiality of BM cells not fully examined
Hayashi et al. (2008) (18)	Mesenchyme stem cells (MSCs)	–	Mice	Inject MSCs or vehicle into gastric wall surrounding acetic acid-induced ulcer	Transplantation significantly accelerated gastric ulcer healing compared with that observed in the control group	Acetic acid-induced ulcers differ from refractory gastric ulcers
Xia et al. (2019) (19)	Adipose-derived mesenchyme stem cells (ADMSCs)	–	Porcine	Inject ADMSCs into NSAID-induced gastric ulcers	Enhanced reepithelization and neovascularization at day 7 and day 21 compared with what was observed in the control group	Very short follow-up time
Zhao et al. (2021) (20)	–	Polyurethane/small intestinal submucosa (PU/SIS) hydrogel	Canine	Deliver PU/SIS hydrogel endoscopically to the ESD-induced ulcer site	Significantly accelerated healing at the early stages Complete healing observed at 4 weeks	Mechanical properties of PU/SIS hydrogel not determined
Full-thickness defects						
Hori et al. (2002) (21)	–	Collagen scaffold graft	Canine	Anterior wall of stomach replaced with collagen sponge scaffold of 4 cm× 4 cm and analyzed 16 weeks later	Regeneration of proton pump and thin muscle layer observed	ACh-induced contraction not observed
Araki et al. (2009) (22)	–	Collagen with a biodegradable copolymer (“new sheet”)	Canine	New-sheet was sutured to repair the 5 cm circular gastric defect	Mucosal side of new-sheet had strength almost equivalent to mucosa of the esophagus; smaller ulcer evident at 16 weeks	Nil smooth muscle layer and regenerated stomach shrank by 60%–80% of its original size
Sirbu-Boeti et al. (2009) (23)	Mesenchyme stem cells (MSCs)	Collagen-agarose scaffold	Rats	MSCs enriched, 3D patches were sutured to 5 mm–7 mm diameter anterior stomach wall defects	Full regeneration of all four layers of the stomach wall observed at 48 days	MSCs are difficult to harvest
Nakatsu et al. (2015) (24)	Mesenchyme stem cells (MSCs)	Small intestinal submucosa (SIS)	Rats	MSC-seeded SIS was used to repair a 1 cm whole-layer stomach defect	Well-structured smooth muscle layers developed	Study was focused only on smooth muscle layers
Long-segment circumferential defects						
Maemura et al. (2004) (25)	Stomach epithelium organoid unit	Biodegradable polymer scaffold	Rats	Organoids-on-polymer units implanted into omenta of recipient adult rats and anastomosed after gastrectomy	A well-developed neostomach formed at 24 weeks	Limited applicability to large animals or humans

"-" means not applicable.

**Table 5:** Summary of *in vivo* animal studies on intestinal tissue engineering.

Author (year)	Cell source	Scaffold	Animal model	Methodology	Outcome	Limitations
Partial-thickness defects						
Keane et al. (2017) (26)	–	ECM hydrogel (ECMH)	Rats	Administer ECMH to rats with ulcerative colitis (UC) via enema	ECMH significantly reduced the clinical and histologic severity of UC at 7 days and 14 days	Only one UC animal model used
Yui et al. (2012) (27)	Colon organoids formed from Lgr5^+^ cells	–	Rats	Transplant colon organoids into superficially damaged mouse colon	Donor-derived cells constituted a single-layered epithelium with self-renewing crypts at 4 weeks	Further optimization of organoids formation is required
Watanabe et al. (2022) (28)	Epithelial organoids	–	Mice	Infuse epithelial organoids into the luminal space via the anus in a mouse model of colitis	Macroscopically full epithelialization occurred 2 weeks after transplantation	Successful engraftment rate was only 46.5%. Only one UC animal model was used
Full-thickness defects						
Chen et al. (2001) (29)	–	Small intestinal submucosa (SIS)	Canine	Repair a 7 cm × 3 cm full-thickness defect on the small intestinal wall with a SIS patch	Mucosa, submucosa, smooth muscle, and serosa regenerated in the newly formed bowel wall	Poorly organized submucosa and smooth muscle layer
Nakase et al. (2006) (30)	Autologous smooth muscle cells (SMC)	Collagen scaffold	Canine	Repair a 1 cm × 1 cm full-thickness defect on the ileum with SMC- loaded collagen sponge and silicone sheet	Developed full epithelial layer with numerous villi and smooth muscle layer at 12 weeks	Very small defect size
Long-segment circumferential defects						
Grikscheit et al. (2004) (31)	Small intestine organoid units	Polymer scaffolds	Rats	Organoids-on-polymer units implanted into rats’ omenta to form tissue-engineered small intestine (TESI) that is later transplanted to rats that underwent massive small bowel resection	Regenerated intact epithelial, muscular, vascular, and neural components. Accelerated weight gain and B12 serum level in rats receiving a TESI	Potential immunogenicity to donor cell source
Meran et al. (2020) (32)	Human derived organoid units	Human intestinal scaffolds	Rats	Human-derived organoids-on-polymer units placed into bioreactor and then into rats’ omenta	Human-derived TESI retained jejunal epithelial identity, showing some enzymatic activity and barrier function	TESI did not fully recapitulate a mature crypt–villus morphology and was enterocyte-dominant

"-" means not applicable.

**Table 6:** Summary of human studies on rectal and anal tissue engineering.

Author (year)	Type of fistula(s)	Cell source ± scaffold	Patient number	Methodology	Outcome	Limitations
Full-thickness defect—stem cell therapy						
Garcia-Olmo et al. (2009) (33)	Complex perianal fistulas - cryptoglandular (*n* = 35) and Crohn’s disease (*n* = 14)	Expanded adipose-derived stem cells (ASCs)	49	Intralesional injection of fibrin glue or fibrin glue with 20 million ASCs after fistula curettage and closure of internal opening	Fistula healing in 71% of ASC patients, compared with only 16% in the control group; higher quality-of-life scores in patients receiving ASCs	Significant clinical costs that are difficult to evaluate
Herreros et al. (2012) (34)	Complex cryptoglandular perianal fistulas	Autologous adipose-derived stem cells (ASCs)	183	A total of 64 patients received 20 million ASCs (Group A); 60 received ASCs with fibrin glue (Group B); and 59 received fibrin glue only (Group C)	At 24 to 26 weeks, healing rates of 54.55%, 83.33%, and 18.18% were observed in group A, B and C; MSCs with fibrin glue considered a safe and efficacious treatment of complex fistula-in-ano	27% loss of sample size in analysis (attrition bias)
Panes et al. (2016) (35)	Complex perianal fistulas in Crohn’s disease	Darvadstrocel Cx601 [allogeneic, expanded, adipose-derived stem cells (ASCs)]	212	Patients randomized to receive a single intralesional injection of 120 million Cx601 cells (*n* = 107) or placebo (*n* = 105)	Combined remission achieved in 50% of patients receiving Cx601, compared with 34% of those receiving a placebo treatment	Exclusion of younger patients and fistulas having multiple external openings
Topal et al. (2019) (36)	Complex non-Crohn’s disease perianal fistulas	Autologous adipose-derived stem cells (ASCs)	10	Intralesional injection of autologous ASCs after fistula curettage and closure of internal opening	A healing rate of 70% at the 30-day follow-up, 80% at the 90-day follow-up, and 70% at the 9-month follow-up	Lack of a control group and variable ASCs dose
Dige et al. (2019) (37)	Complex perianal fistulas in Crohn’s disease	Freshly collected autologous adipose tissue	21	Intralesional injection of freshly collected autologous adipose tissue after fistula curettage and closure of internal opening	After 6 months, overall response in 76% of patients, with complete fistula healing in 57% of patients, ceased secretion in 14% of patients, and reduced secretion in 5% of patients	Lack of a control group; unblinded clinical follow-up
Garcia-Arranz et al. (2020) (38)	Complex cryptoglandular perianal fistulas	Autologous adipose-derived stem cells (ASCs)	44	A total of 23 patients received 100 million ASCs with fibrin glue and 21 patients received fibrin glue only	At 2 years, 50% of patients in the treatment group achieved remission, compared with 26.3% in the control group	High dropout rates
Laureti et al. (2020) (39)	Complex perianal fistulas in Crohn’s disease	Autologous micro-fragmented adipose tissue	15	Intralesional injection of autologous micro-fragmented adipose tissue prepared using the Lipogems technique	At 24 weeks, 10 out of 15 patients achieved combined remission (both clinical and radiographic improvement), four patients showed improvements, and treatment was unsuccessful in one patient	Small sample size and lack of a control group
Barnhoorn et al. (2020) (40)	Complex perianal fistulas in Crohn’s disease	Allogeneic bone marrow-derived mesenchymal stromal cells (bmMSCs)	21 (13 for long-term follow-up)	Intralesional injection of allogeneic bmMSCs after fistula curettage and closure of internal opening	High rate of fistula closure maintained 4 years post treatment, with no patients developing anti-HLA antibodies	Small sample size for long-term follow-up
Ascanelli et al. (2021) (41)	Complex cryptoglandular perianal fistulas	Autologous centrifuged adipose tissue containing progenitor cells	116	Patients randomly allocated to receive intralesional injection of autologous adipose tissue or with fistula surgery	At 4 weeks, the healing rate was 63.8% of healing rate in the experimental group and 15.5% in the control group; lower postoperative pain in the experimental group	Absence of blinding; lack of standardization of stem cell contents
Cabalzar-Wondberg et al. (2021) (42)	Complex perianal fistulas in Crohn’s disease	Allogeneic adipose-derived stem cells (ASCs)	11	Intralesional injection of 120 million allogeneic ASCs after fistula curettage and closure of internal opening	After 41.5 weeks, complete fistula closure was observed in 72.7% (8 out of 11) of patients	Small sample size; radiograph healing not assessed due to cost issues
Maciel Gutierrez et al. (2021) (43)	Complex non-Crohn’s disease perianal fistulas	Allogeneic adipose tissue-derived mesenchymal stem cells (ADMSCs)	20	Intralesional injection of 40 million allogeneic ADMSCs after fistula curettage and closure of internal opening	At 8 weeks, complete closure of the complex anal fistula was achieved in 69% of ADMSC-treated patients	Non-randomized; absence of a control group
Guillo et al. (2022) (44)	Complex perianal fistulas in Crohn’s disease	Autologous adipose-derived stromal vascular fraction (ADSVF) with microfat	10	Intralesional injection of ADSVF and microfat after fistula curettage and closure of internal opening	At 3 years post injection, combined remission was achieved in 70% of the patients with a significant improvement in the MAGNIFI-CD MRI score	Small sample size; retrospective design; lack of a control group
Lightner et al. (2023) (45)	Complex perianal fistulas in Crohn’s disease	*Ex vivo* expanded allogeneic bone marrow-derived mesenchymal stem cells (bmMSCs)	23	Intralesional injection of 75 million allogeneic bmMSCs after fistula curettage and closure of internal opening	At 6 months, complete clinical and radiographic healing was observed in 83% of patients in the experimental group and 40% of those in the control group; the perianal Crohn’s disease activity index, Wexner incontinence score, and Van Assche score were all significantly decreased in bmMSC patients	Single-blinded and single institution
Pak et al. (2023) (46)	Complex non-Crohn’s disease perianal fistulas	Human placental mesenchymal stem cells (MSCs)-derived exosomes	11	Intralesional injection of MSCs-derived exosomes after fistula curettage and closure of internal opening	A total of 10 out of 11 patients showed clinical improvement, including five who showed complete resolution	Small sample size with possible selection bias; lack of a control group
Furukawa et al. (2023) (47)	Complex perianal fistulas in Crohn’s disease	Darvadstrocel Cx601 (allogeneic, expanded, adipose-derived stem cells [ASCs])	22	Intralesional injection of Cx601 (120 million cells) after fistula curettage and closure of internal opening	At week 24, 59.1% of patients achieved combined remission; at week 52, 68.2% of patients achieved combined remission	Small sample size; open-label study with no comparator
Full-thickness defect—stem cell therapy with bioscaffold						
Dietz et al. (2017) (48)	Complex perianal fistulas in Crohn’s disease	Autologous mesenchymal stem cell-coated fistula plug (MSC-MATRIX)	12	Draw MSC-MATRIX fistula plugs through the fistula tract and suture in place	At 6 months, complete clinical healing and radiographic markers of response were seen in 83% (10 out of 12) of patients	Lack of control a group; small sample size; short follow-up period
Dozois et al. (2019) (49)	Trans-sphincteric cryptoglandular perianal fistulas	Autologous mesenchymal stem cell-coated fistula plug (MSC-MATRIX)	15	Draw MSC-MATRIX fistula plugs through the fistula tract and suture in place	At 6 months, three patients showed complete clinical healing, eight showed partial healing, and four showed no clinical improvement; radiographic improvement was observed in 11 out of 15 patients	Lack of a control group; small sample size; short follow-up period

## Discussion

### Esophageal tissue engineering

#### Partial-thickness defects

Partial-thickness defects of the esophagus commonly occur as a result of removing the dysplastic mucosa and/or superficial cancer tissue following ESD. If >50% of the mucosal circumference is resected, up to 70% of patients will develop fibrosis and strictures (50). Current methods to prevent post-ESD injury are limited, warranting novel approaches to stimulate mucosal regeneration. Tissue-engineering techniques such as cell therapy, cell sheet, and bioscaffolds have been studied in various animal models (**Table 2**) and have shown promising results in re-epithelializing the partial-thickness defects of the esophagus and reducing stricture formation (1, 5–7).

In 2010, Honda et al. carried out endoscopic injections of adipose tissue-derived stromal cells (ADSCs) into the submucosa following circumferential esophageal ESD in five dogs (5). Compared with the proportion of luminal narrowing in the control group (76%), the experimental group demonstrated a significant reduction in luminal constriction (45%). In a related context, Ohki et al. in 2006 conducted endoscopic transplantation of autologous oral mucosa epithelial cell sheets following semi-circumferential ESD in dogs (1). The cell sheets securely adhered to the wound site, effectively enhanced wound healing, and prevented the development of strictures and stenosis. Similar results were also observed by Kanai et al. in 2012 using autologous epidermal cell sheets, and by Nieponice et al. in 2009 in their application of dermal extracellular matrixes (6, 7).

**Figure 2:**
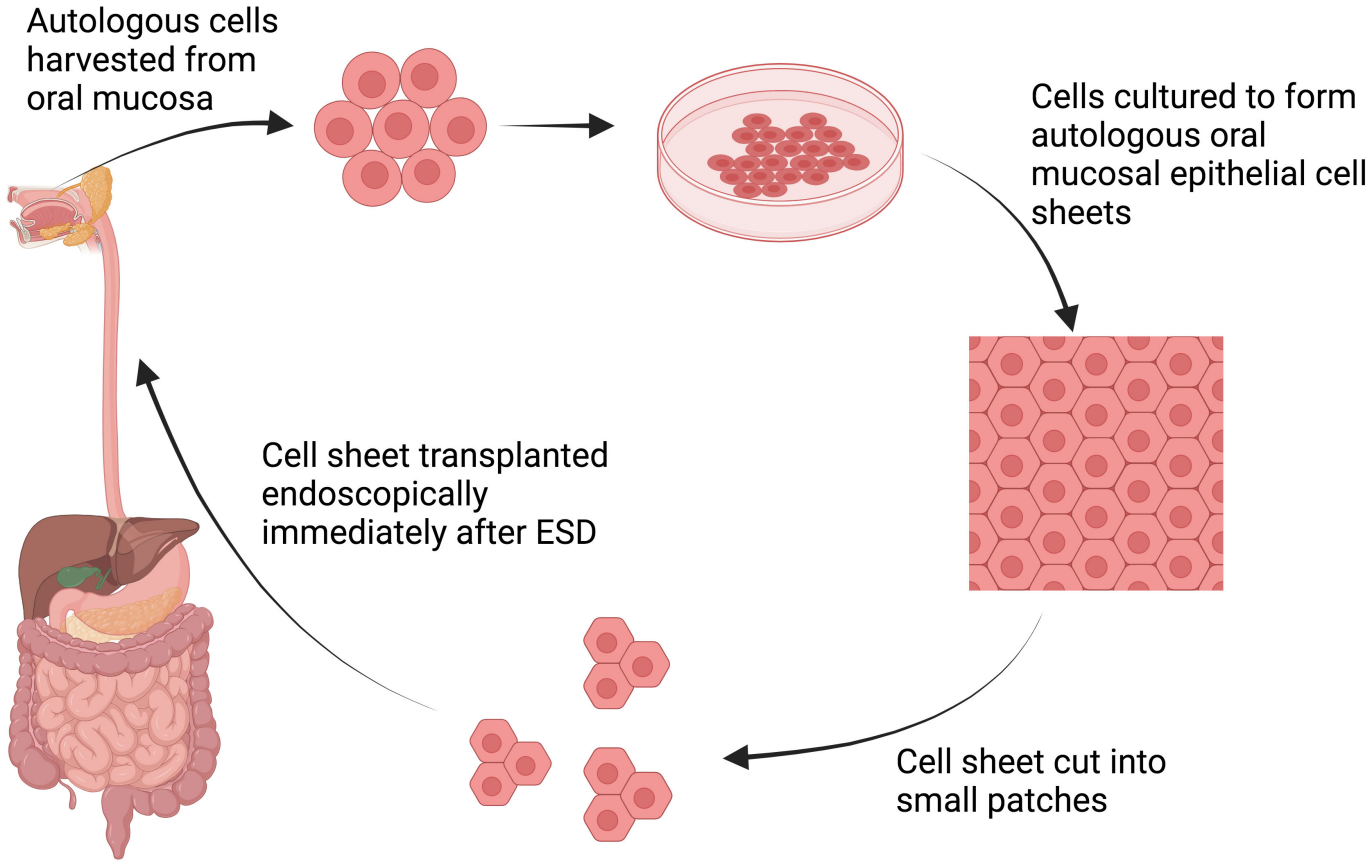
Schematic explanation of cell sheet tissue engineering. Autologous cells are harvested from oral mucosa and subsequently cultured to form autologous oral mucosa epithelial cell sheets. The manufactured cell sheet is then cut into small patches, allowing endoscopic transplantation immediately after ESD procedures (13). Created with BioRender.com.

In 2021, Ohki et al. attempted the first human clinical application of tissue-engineered cell sheet technology in an open-label, single-arm, single-institute study (**Table 3**) (13). Specimens of oral mucosal tissue were collected from nine patients with superficial esophageal cancer. Epithelial cell sheets were fabricated *ex vivo* from the harvested specimens. These autologous oral mucosal cell sheets were later transplanted endoscopically to the ulcer surfaces of patients who had undergone ESD of >50% of the circumference (**Figure 2**). These cell sheets easily adhered to the ESD site and completely re-epithelized within a median time of 3.5 weeks. With only one exception, no patients developed esophageal strictures, dysphagia, or other serious complications following the procedure.

Although promising, this approach has limited practical utility as many centers do not have the expertise needed to fabricate tissue-engineered cell sheets. Hence, in 2017 Yamaguchi et al. studied the feasibility of endoscopic transplantation of oral mucosal cell sheets that had been manufactured and transported from a distant site 1,200 km away (12). In this experiment, 10 patients underwent complete circular or semicircular ESD for superficial esophageal neoplasms and were subsequently engrafted with cell sheets. Following cell sheet transplantation, re-epithelialization occurred within a median of 5 weeks without stricture formation.

#### Full-thickness defects

**Figure 3:**
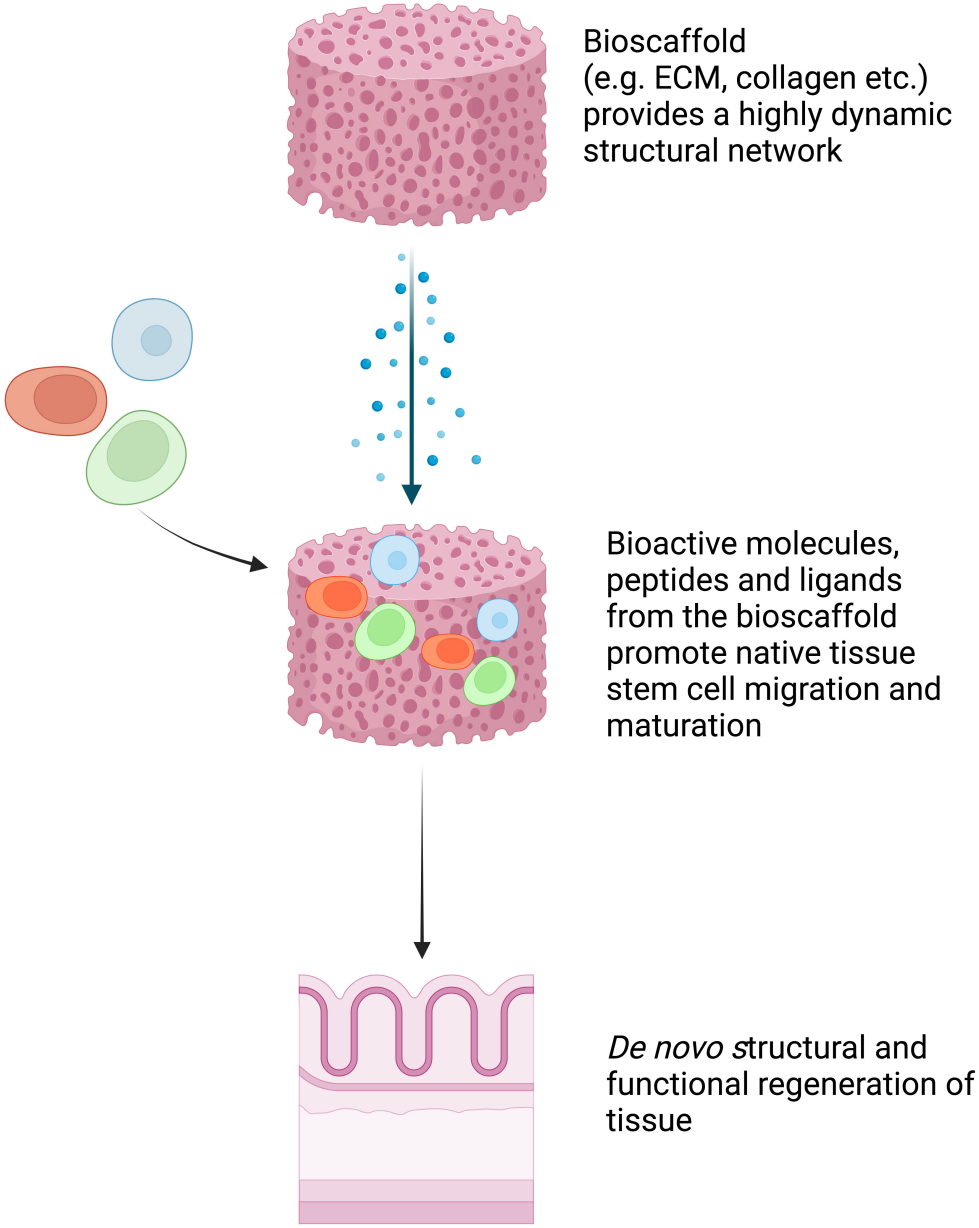
Schematic explanation of bioscaffold tissue engineering. Bioscaffold tissue provides a highly dynamic structural network that promotes native tissue stem cell migration and maturation into site-specific phenotypic cells. It facilitates this process by releasing bioactive molecules, peptides, and ligands that provide signals to local and migrant stem cells to induce the *de novo* structural and functional regeneration of tissue (8). Created with BioRender.com.

Full-thickness patch defects of the esophagus, such as perforations, leaks, and fistulas, can occur spontaneously, as seen in Boerhaave syndrome, or as complications after certain procedures. These types of defects often result in esophageal distortion and, thus, to maintain the tubular structure of the esophagus during regeneration, the majority of studies have used extracellular matrices (ECMs) with removable stents (8). The native ECMs are derived from human or animal tissues after removal of the cellular and immunogenic components (2, 8). They maintain a highly dynamic structural network that promotes native tissue stem cell migration and maturation into site-specific phenotypic cells (8). ECMs can facilitate this process by releasing bioactive molecules, peptides, and ligands that provide signals to local and migrant stem cells to induce the *de novo* structural and functional regeneration of tissue (**Figure 3**). In 2000, Badylak et al. showed successful re-epithelialization with full esophageal wall regeneration at day 50 after applying xenogeneic ECM patches to full-thickness defects in dogs (8).

By applying similar principles, in 2014 Nieponice et al. conducted the first human study by using porcine urinary bladder ECMs as reconstructive patches to repair large, full-thickness defects in four patients (**Table 3**) (2). The ECM patches were sutured to the edges of the esophageal full-thickness defects. Clinical outcomes were favorable in all cases, with complete mucosal remodeling and normal epithelialization observed at 2 months. The immediate foreseeable application of this bioscaffold technology is to repair the large perforations, leaks, and fistulas that are not manageable with currently available endotherapy. More clinical trials, however, will be required to validate the results from this first human study.

#### Long-segment circumferential defects

Long-segment circumferential defects can occur in congenital esophageal atresia or post esophagectomy for either benign or malignant conditions. Significant morbidities and poor quality of life are associated with these types of defects. Animal studies using acellular allogeneic or xenogenic ECM alone failed to regenerate a circumferential, long-segment esophagus (9). This suggests that there are limitations to the size of defect that may be replaced using the ECM methodology. Instead, animal studies (**Table 2**) using an ECM cellularized with pluripotent cells either in a bioreactor or *in vivo* successfully achieved *de novo* organogenesis of the esophagus (9, 10). In 2017, Catry et al. carried out a 3 cm circumferential replacement of the abdominal esophagus in 10 mini pigs using an MSC-seeded matrix and compared its efficacy with that of a matrix alone, carried out in a control group (9). The graft area was covered with an esophageal removable stent. On day 45, all MSC group specimens exhibited mature squamous epithelia covering the entire graft area, whereas none of the control group specimens achieved this until day 95. In a more recent study published in 2022, Sundaram et al. successfully regrew the full-layered esophagus in a porcine model using the Cellspan Esophageal Implant™ (CEI), a polyurethane tubular mesh cell delivery device (Cellframe™ Technology) seeded with autologous ADMSCs (12). Furthermore, the extended 1-year follow-up period revealed the successful restoration of oral nutrition, normal animal growth, and the overall safety of this therapeutic approach.

By applying this principle, in 2016 Dua et al. reported the first human case of *de novo* regeneration of the esophagus in a 24-year-old patient (**Table 3**) (4). Through a large paraspinal abscess, a 5 cm full-thickness circumferential defect was formed in the upper esophagus, resulting in direct communication between the pharynx and mediastinum. Three self-expanding metal stents (SEMSs) were first placed endoscopically to maintain the tubular configuration of the esophagus. A commercially available ECM (AlloDerm™) was then placed around the stent and sprayed with autologous platelet-rich plasma. The platelet-derived growth factor (PDGF) from the autologous platelet-rich plasma assisted the migration of endogenous pluripotent cells in the patient’s thoracic cavity, which served as an *in vivo* bioreactor. The SEMSs were removed 3.5 years after their placement, and it was observed that the esophagus had regenerated to full thickness and that it contained a stratified squamous epithelium, a normal five-layer wall, and peristaltic motility with bolus transit. In a more recent case report published in 2021, Aho et al. successfully implanted the Cellspan Esophageal Implant™ (CEI) in a 75-year-old patient to replace a 4 cm full circumferential segment of esophagus that had been invaded by a tumor (15). Complete luminal epithelialization and partial esophageal tissue regeneration were observed after 7.5 months.

These results, from both case reports, hold significant promise. A biocompatible interposition graft that aids in esophageal regeneration holds significant clinical potential, particularly in the context of short-circumferential segment resection (15). Further clinical trials are warranted to validate these findings and potentially simplify the technique, which currently relies on practitioner(s) having specialized expertise (4, 15).

### Stomach tissue engineering

The principles used in stomach tissue engineering closely resemble those utilized in esophageal tissue engineering. Some promising results have been obtained in animal models, but, as of now, there have been no published human studies in the field of stomach tissue engineering.

#### Partial-thickness defects

Partial-thickness defects of the stomach can occur in peptic ulcer disease or as a result of ESD for the removal of superficial gastric Neoplasms. Both cell therapy and bioscaffold techniques have achieved success in promoting the healing of this type of defect in animal models (**Table 4**).

In 2008, Askarov et al. conducted a study in which precultured multipotent mesenchymal stromal cells (MMSCs) from autologous bone narrow were injected into the site of chronic gastric ulcers (5 mm–7 mm in diameter) in rats (16). This treatment resulted in a significant reduction in the ulcer defect area and accelerated epithelialization on days 10 and 20 compared with what was observed in the control group. On day 30, full epithelialization of the gastric mucosa was observed in the MMSC transplantation group. In 2008, Hayashi et al. and Nishida et al. observed similar results in their experiment on the topical transplantation of MMSCs to gastric ulcers in mice (17, 18). It was suggested that MMSCs delivered to the zone of the ulcer defect induce regeneration by releasing regulatory peptides, immunocytokines, and growth factors, in turn promoting neoangiogenesis and ulcer healing (17).

In a more recent study published in 2019, Xia et al. endoscopically applied adipose-derived mesenchymal stem cells (ADMSCs) to non-steroidal anti-inflammatory drug (NSAID)-related gastric ulcers in a porcine model (19). This approach resulted in the model exhibiting reduced inflammatory infiltration, enhanced re-epithelization, and neovascularization at both day 7 and day 21 compared with what was observed in the control group (which was injected with saline). However, it was observed that only a small proportion of engrafted ADMSCs displayed myofibroblast and epithelial cell characteristics *in vivo*, indicating that the healing process of the ulcer may be less dependent on stem cell transdifferentiation. Subsequent experiments involving the submucosal injection of MSC-derived secretome demonstrated a therapeutic effectiveness that was on par with stem cell transplantation. This indicates that the paracrine action of stem cells plays a central role in the healing process.

As an alternative to cell therapy, in 2021 Zhao et al. developed a polyurethane/small intestinal submucosa (PU/SIS) hydrogel treatment with high levels of biocompatibility, bioadhesion, and pH sensitivity (20). This modified ECM was delivered endoscopically to the ESD-induced ulcer site (3 cm in diameter) in a canine model. The PU/SIS hydrogel treatment was shown to significantly accelerate healing at the early stages compared with that observed in the control group (which received proton pump inhibitors). At 4 weeks, the ulcer was completely healed in the PU/SIS group, but complete healing took 7 weeks in the control group. It was suggested that this modified ECM provided a protective microenvironment that released growth factors, in turn promoting ulcer healing. However, more animal studies are recommended to validate the results obtained in these studies before these technologies are extrapolated to humans.

#### Full-thickness defects

For full-thickness patch defects of the stomach, both acellular and cellular tissue-engineering approaches have been reported in animal studies (**Table 4**). In terms of acellular approaches, in 2002 Hori et al. established a method for *in situ* stomach tissue engineering in a canine model by suturing an acellular collagen scaffold graft to a 4 cm × 4 cm anterior stomach wall defect (21). A silicone sheet was used as a patch on the luminal side to protect the scaffold from degradation by digestive juice. At 16 weeks, the stomach wall had regenerated, and a gastric mucosa covered the entire lesion. However, the silicone sheet presented technical difficulties for suturing and needed to be removed endoscopically after healing, and therefore has limited clinical usability. To avoid the need for endoscopic removal, in 2009 Araki et al. experimented with single-sheet collagen scaffolds reinforced with a biodegradable copolymer (22). This new scaffold was sutured to repair a 5 cm circular full-thickness defect on the anterior stomach in dog models. At 16 weeks, the serosal side of the implanted scaffold was fully covered by omentum, with mucosa regeneration observed on the luminal side. However, regeneration of the smooth muscle layer did not occur, and early shrinkage of the implanted scaffold was also observed.

Regarding cellular approaches, in 2009 Sirbu-Boeti et al. seeded a collagen-agarose scaffold with mesenchymal stem cells (MSCs) obtained from bone marrow (23). The MSC-enriched patches were sutured to anterior stomach wall defects (5 mm–7 mm in diameter) in rats. The full regeneration of all four layers of the stomach wall was observed at 48 days. In 2015, Nakatsu et al. demonstrated similar results by grafting MSC-seeded small intestinal submucosa to a 1 cm whole-layer stomach defect in a rat model (24). This combination of bioscaffold and cell therapy enabled the effective regeneration of the stomach wall both histologically and functionally.

#### Long-segment circumferential defects

**Figure 4:**
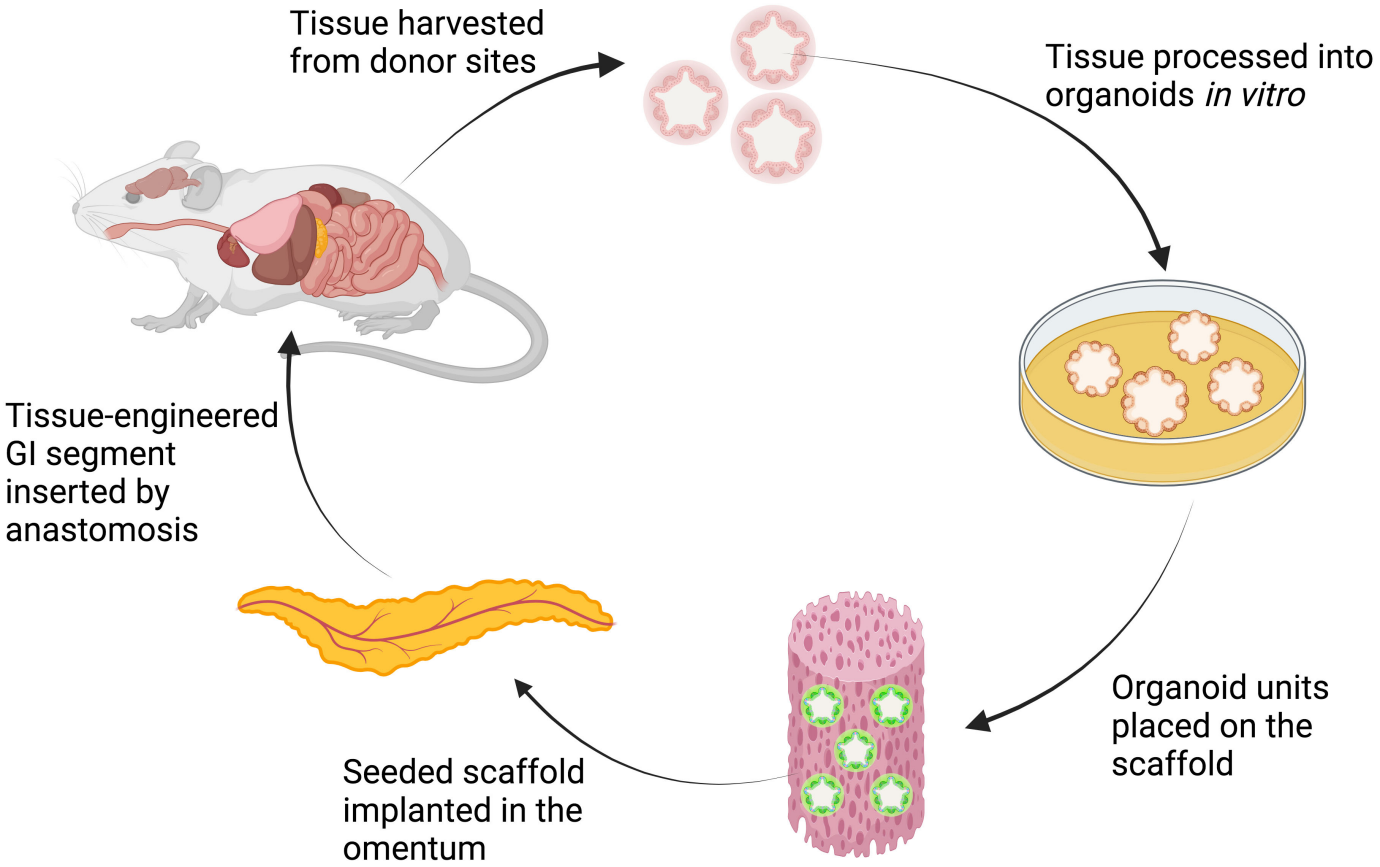
Schematic explanation of organoids-on-polymer tissue engineering. The harvested tissue from donor sites is processed *in vitro* to form organoids that contain a mixture of epithelium and mesenchyme. The organoids are then seeded onto the scaffold and implanted in the omentum of the animal. Through *in vivo* organogenesis, tissue-engineered GI segment is then harvested and inserted to replace the defective segment (25, 31). Created with BioRender.com.

To repair whole-layer circumferential defects of the stomach, animal studies (**Table 4**) successfully generated tissue-engineered stomach by implanting bioscaffolds loaded with pluripotent cells or organoids into the animals’ peritoneal cavities (**Figure 4**) (25). The stomach organoids were derived from gastric stem cells and contained a mixture of epithelia and mesenchymes. The mesenchymal–epithelial interactions in the organoids are crucial to the differentiation and morphogenesis of the engineered tissue (25).

In 2004, Maemura et al. seeded stomach epithelium organoids to the biodegradable polymer tube and wrapped them in the omenta of adult Lewis rats (25). The organoid polymer constructs formed cyst-like tissue-engineered stomachs in 3–6 weeks. In rats that underwent gastrectomies, the autologous tissue-engineered stomach was then harvested and anastomosed to the esophagus and small intestine. The omentum attached to the tissue-engineered stomach was retained to maintain the blood supply to the stomach. At 24 weeks, a well-developed neostomach was observed, with a continuous mucosa lining the lumen and stratified, smooth muscle-like layers. Neither stenosis nor obstruction was observed at the anastomosis site. These findings suggest that the tissue-engineered stomach had functionally normal secretion and motility, similar to those of a native stomach. Nevertheless, this methodology appears to have limited applicability in large-animal models or in humans due to the tissue-engineered stomach needing to be formed in the abdominal cavity of the recipient. Furthermore, long-term follow-up studies are required to demonstrate the functionality and safety of tissue-engineered stomachs.

### Intestinal tissue engineering

#### Partial-thickness defects

The intestinal epithelium layer is often damaged by various gastrointestinal diseases such as inflammatory bowel diseases (IBD). Animal studies seeking to address this issue have used both ECM bioscaffold and intestinal organoids to repair intestines with significant mucosal damage (**Table 5**).

In 2017 Keane et al. prepared an ECM hydrogel (ECMH) from a porcine small intestine submucosa and applied it to rats with ulcerative colitis via enema (26). The ECMH adhered to the colonic tissue and significantly reduced the clinical and histologic severity of the disease in the hydrogel-treated rats at days 7 and 14 compared with that in the control rats. This demonstrated the effect of ECMH treatment in restoring colonic epithelial barrier function.

On the other hand, in 2012 Yui et al. used single stem cells from a mouse colon to form colon organoids *in vitro* and transplanted them into superficially damaged mouse colon (27). The transplanted organoids colonized injured mucosa and formed functional donor-derived epithelium 4 weeks post transplantation. After long-term follow-up of more than 6 months, the graft remained stable and showed evidence of mucosal proliferation and renewal. These findings were supported by a study from Watanabe et al. in 2022, which showed similar results (28). The therapeutic application of ECMH or organoid culture to generate new intestinal tissue could provide a novel mechanism to restore barrier function in IBD, especially improving the prognosis of those with refractory ulcerative colitis.

#### Full-thickness defects

Different bioscaffolds or cell sources have been used to repair full-thickness intestinal defects in various animal models (**Table 5**). In 2001 Chen et al. repaired a 7 cm × 3 cm full-thickness defect on the small intestinal wall with a small intestinal submucosa patch in a canine model (29). A histologic evaluation showed the presence of mucosa, submucosa, smooth muscle, and serosa in the newly formed bowel wall. However, the architecture of the submucosa and smooth muscle layers were not well organized. To improve this, in 2006 Nakase et al. used collagen scaffolds seeded with autologous smooth muscle cells (SMC) before implanting them to full-thickness ileal defects in dogs (30). At 12 weeks, a well-developed epithelial layer with villous structures and an organized smooth muscle layer was observed.

#### Long-segment circumferential defects

The ultimate goal in intestinal tissue engineering, however, is to generate a whole-layer circumferential intestinal graft for transplantation, as it may offer curative therapy for patients with short bowel syndrome. In 2004, Grikscheit et al. created tissue-engineered small intestines (TESI) through the transplantation of organoid units on a polymer scaffold before implanting it into the omentum of a rat (**Figure 4**) (31). The harvested TESI was then implanted to five rats that had underwent massive small bowel resection. The immunohistochemical studies of the neo-intestine revealed intact epithelial, muscular, vascular, and neural components. The postoperative weights and B_12_ absorption abilities of animals receiving the TESI were significantly improved compared with those animals that had the bowel resection alone, indicating that the TESI functioned *in vivo* to meet basic physiologic needs. However, further investigation is required to study the potential immunogenicity of the TESI.

To bring this organoid-on-scaffold approach a step closer to clinical practice, in 2020 Meran et al. generated the first patient-derived TESI with organoids and bioscaffolds obtained from children with intestinal failure (32). Patient-derived organoids were seeded into the decellurized human intestine matrix before being transferred to a bioreactor system for 11 days. The patient-derived TESI was then transplanted into immunodeficient mice for 1 week. Serial histology analyses showed that the patient-derived TESI retained jejunal epithelial identity, but that it did not fully recapitulate a mature crypt–villus morphology and was enterocyte-dominant. Future studies are needed to further differentiate patient-derived TESI into fully functional jejunum *in vivo*.

### Rectal and anal tissue engineering

#### Full-thickness defects

Perianal fistula is a type of full-thickness defect with a tract between the anorectal canal and the perianal skin. It is one of the most common colorectal diseases, with an incidence of 1–8 per 10,000 individuals, and with up to 25% of cases associated with Crohn’s disease (51). Although most simple perianal fistulas can be cured by surgical operation, the success rate of surgeries on complex and refractory fistulas is low, and such surgeries are also associated with a high rate of complications. These complications, such as fistula recurrence and fecal incontinence due to sphincter and perianal tissue destruction, can significantly impact on patients’ quality of life and result in high healthcare costs (51). Recently, tissue engineering techniques such as the local injection of mesenchymal stem cells (MSCs) have shown great promise in treating complex perianal fistulas and have a minimal risk of fecal incontinence. A number of human studies have now demonstrated the safety and efficacy of this novel therapy (**Table 6**) (33–49).

In 2016, Panes et al. conducted a well-known phase 3 randomized, double-blind controlled trial in Europe and Israel (ADMIRE-CD study) to assess the safety and efficacy of darvadstrocel Cx601 (a commercially available allogeneic adipose-derived stem cells preparation) for treating complex perianal fistulas in patients with Crohn’s disease who had not responded to conventional or biological treatments (35). Darvadstrocel Cx601 was administered by direct injection after curettage and primary closure of the fistula tract. At week 24, 50% of patients receiving darvadstrocel Cx601 demonstrated the clinically confirmed closure of all treated external openings and the absence of collections, and this effect was observed to have been maintained at the week 52 follow-up. Similar results were also observed in 2023 by Furukawa et al. in a phase 3 study on the use of darvadstrocel Cx601 in a Japanese patient population with refractory perianal fistulizing Crohn’s disease (47).

In 2020, Barnhoorn et al. evaluated the long-term safety and efficacy of allogeneic bone marrow-derived mesenchymal stromal cell (bmMSC) therapy in a patient with Crohn’s disease-associated perianal fistulas (40). After 4 years of follow-up, they observed the sustained high rate of fistula closure and noted a significant improvement in the quality of life reported by the bmMSC-treated patients. In addition, none of the patients who underwent bmMSC therapy developed anti-HLA antibodies within either the initial 24-week period or the extended 4-year time frame. In contrast, Panes et al. observed that 34% (18 out of 53) of patients who received Cx601 therapy and who were initially negative for anti-HLA class I antibodies developed these antibodies following MSC treatment (35). This difference in anti-HLA antibody formation may be attributed to the origin of the MSC product. Although the clinical significance of the presence of anti-HLA antibodies remains unclear, it is important to note that the current studies have not raised any safety or efficacy concerns (35, 40). Several studies further support the efficacy and safety of local MSCs therapy in refractory Crohn’s disease fistula (see **Table 6**) (33, 37, 39, 42, 44, 45, 48).

It is believed that MSCs exert immunosuppressive effects and modulate the functions of different immune cells, resulting in their exerting disease-modifying and healing effects in Crohn’s disease (35). Moreover, the absence of histocompatibility complex class 2 and the limited expression of human leukocyte antigen (HLA) class 1 in human MSCs have established their suitability for safe allogenic transplantation (35, 40, 47). This quality renders commercially available MSC preparations (such as darvadstrocel) a viable and pragmatic choice when transitioning the technique from the laboratory to real-world applications.

Satisfactory therapeutic outcomes were similarly evident with local MSC transplantation in the treatment of complex perianal fistula in non-Crohn’s-disease cases. In 2021, Ascanelli et al. administered autologous centrifuged adipose tissue containing progenitor cells to 58 patients with complex cryptoglandular anal fistulas (41). Their study revealed that 63.8% of patients who received this stem cell therapy achieved clinical healing within 4 weeks, and that 86.2% had achieved complete healing at the 6-month mark. In addition, the injection of adipose tissue-derived stem cells (ADSCs) significantly reduced postoperative anal pain and allowed an early return to daily activities for the patients. Similarly, in 2020 Garcia-Arranz et al. also observed long-term and sustained fistula healing following ADSCs therapy for the treatment of cryptoglandular fistula, with the healing rate maintained at 50% in the treatment group (38). Several other studies have provided additional evidence of the efficacy and safety of MSCs transplantation in non-Crohn’s-disease complex perianal fistula (**Table 6**) (33, 34, 36, 43, 46).

To further increase the remission rate, another novel approach has been devised, which entails the synergistic combination of MSCs with bioscaffolds to enhance cell retention at the site of injury. In 2017, Dietz et al. applied autologous MSC-loaded fistula plugs (MSC-MATRIX) to patients with refractory Crohn’s disease fistulas (48). At 6 months, 10 out of 12 patients (83%) exhibited complete clinical healing and radiographic response. In 2019, Dozois et al. conducted an additional evaluation of the safety and efficacy of MSC-MATRIX in patients with trans-sphincteric cryptoglandular fistulas (49). At 6 months, 11 out of 15 patients (73%) exhibited radiographic improvement, consisting of three patients who achieved complete clinical healing and eight who showed partial healing. These data collectively demonstrate the therapeutic potential for MSC-MATRIX in treating refractory diseases. However, both studies are limited by their small patient cohorts and short follow-up durations (48, 49). To rigorously assess bioscaffolds loaded with MSCs, it is imperative to conduct a randomized, controlled phase II trial. Furthermore, there are currently no standardized guidelines regarding MSCs source, cell types, or the optimal number of administrations, making cross-study comparison difficult to carry out. Further research is needed to elucidate whether or not these factors can influence treatment efficacy.

## Future perspectives

Major conceptual advances have been made toward GI tissue engineering, especially in animal models. The extrapolation of technologies used in animal studies to humans is still an experimental process that has been attempted only in the fields of esophageal, rectal, and anal tissue engineering. Although the initial results represent a promising start, several challenges must be overcome prior to the standard clinical application of these techniques. Firstly, the bioengineered tissues commonly lack proper connections to the vascular, nervous, and lymphatic systems in the rest of the GIT, posing a substantial challenge to the full integration of the grafts. In addition, scaling up the manufacture of grafts of different sizes remains an obstacle toward clinical translation. Furthermore, there still remain knowledge gaps in understanding the expansion limit and the *in vivo* stability of organoids and different stem cells, which poses potential long-term risks to recipient safety. Additional work, including prospective studies and long-term follow-up in large-animal models and in humans, is required to fully evaluate the *in vivo* safety and physiologic functions of the grafts.

## Conclusion

Repairing different types of defects in the gastrointestinal tract using tissue-engineering technology have been extensively studied in animal models. Technologies such as mesenchymal stem cells, bio-scaffolds, organoids, and cell sheets are the most promising and closer to clinical translation. Collaboration between GI surgery and regenerative medicine is expected to bring novel therapeutic modalities in the future. Although major conceptual advances have been made, there remain several challenges to overcome, such as the upscaling of the manufacturing process, cost, regulation, and *in vivo* safety concerns. Future large-animal studies and clinical trials will be instrumental in furthering development in this field.

## Author contributions

YL: Data curation, Investigation, Methodology, Writing - original draft, Writing - review and editing. LC: Conceptualization, Supervision, Writing - review and editing. MR: Conceptualization, Supervision, Writing - review and editing.

## Appendix

Appendix 1 Ovid MEDINE® ALL search strategy.

**Table:**

Esophageal tissue engineering			Stomach tissue engineering		
#	Searches	Results	#	Searches	Results
1	Esophagus/cy, pa, ph, su, tr [Cytology, Pathology, Physiology, Surgery, Transplantation]	20,536	1	Stomach/cy, pa, ph, su, tr [Cytology, Pathology, Physiology, Surgery, Transplantation]	26,075
2	Esophageal Diseases/in, su, th [Injuries, Surgery, Therapy]	2,516	2	Stomach Diseases/in, su, th [Injuries, Surgery, Therapy]	2,454
3	Esophageal Neoplasms/in, su, th [Injuries, Surgery, Therapy]	24,346	3	Stomach Neoplasms/su, th [Surgery, Therapy]	33,222
4	1 or 2 or 3	44,426	4	Stomach Ulcer/su, th [Surgery, Therapy]	4,499
5	Tissue Engineering/	44,700	5	1 or 2 or 3 or 4	63,729
6	Regenerative Medicine/	8,390	6	Tissue Engineering/	44,700
7	“Cell- and Tissue-Based Therapy”/	9,169	7	Regenerative Medicine/	8,390
8	Cells, Cultured/	563,776	8	“Cell- and Tissue-Based Therapy”/	9,169
9	Organoids/cy, pa, ph, tr [Cytology, Pathology, Physiology, Transplantation]	2,774	9	Cells, Cultured/	563,776
10	Tissue Scaffolds/	29,354	10	Organoids/cy, pa, ph, tr [Cytology, Pathology, Physiology, Transplantation]	2,774
11	Extracellular Matrix/tr [Transplantation]	418	11	Tissue Scaffolds/	29,354
12	Mesenchymal Stem Cell Transplantation/	14,885	12	Extracellular Matrix/tr [Transplantation]	418
13	5 or 6 or 7 or 8 or 9 or 10 or 11 or 12	633,113	13	Mesenchymal Stem Cell Transplantation/	14,885
14	4 and 13	378	14	6 or 7 or 8 or 9 or 10 or 11 or 12 or 13	633,113
15	Limit 14 to (abstracts and English language and yr=“2000-Current”)	**288**	15	5 and 14	333
			16	Limit 15 to (abstracts and English language and yr=“2000-Current”)	**233**

**Table:**

Intestinal Tissue Engineering			Rectal and Anal Tissue Engineering		
#	Searches	Results	#	Searches	Results
1	Intestine, small/cy, pa, ph, su, tr [Cytology, Pathology, Physiology, Surgery, Transplantation]	25,479	1	Rectum/cy, pa, ph, su, tr [Cytology, Pathology, Physiology, Surgery, Transplantation]	20,010
2	Intestinal Diseases/in, su, th [Injuries, Surgery, Therapy]	3,830	2	Anus/in, su, th [Injuries, Surgery, Therapy]	9,584
3	Colon/cy, pa, ph, su, tr [Cytology, Pathology, Physiology, Surgery, Transplantation]	33,603	3	Rectal Fistula/su, th [Surgery, Therapy]	3,168
4	Intestinal mucosa/cy, pa, ph, su, tr [Cytology, Pathology, Physiology, Surgery, Transplantation]	37,097	4	(1 or 2) and 3	798
5	1 or 2 or 3 or 4	88,941	5	Tissue Engineering/	44,700
6	Tissue Engineering/	44,700	6	Regenerative Medicine/	8,390
7	Regenerative Medicine/	8,390	7	“Cell- and Tissue-Based Therapy”/	9,169
8	“Cell- and Tissue-Based Therapy”/	9,169	8	Cells, Cultured/	563,776
9	Cells, Cultured/	563,776	9	Organoids/cy, pa, ph, tr [Cytology, Pathology, Physiology, Transplantation]	2,774
10	Organoids/cy, pa, ph, tr [Cytology, Pathology, Physiology, Transplantation]	2,774	10	Tissue Scaffolds/	29,354
11	Tissue Scaffolds/	29,354	11	Extracellular Matrix/tr [Transplantation]	418
12	Extracellular Matrix/tr [Transplantation]	418	12	Mesenchymal Stem Cell Transplantation/	14,885
13	Mesenchymal Stem Cell Transplantation/	14,885	13	6 or 7 or 8 or 9 or 10 or 11 or 12 or 13	633,113
14	(6 or 7 or 8 or 9 or 10 or 11 or 12 or 13).m_titl.	39,783	14	3 and 14	78
15	5 and 14	283	15	Limit 14 to (abstracts and English language and yr=“2000-Current”)	**68**
16	Limit 15 to (abstracts and English language and yr=“2000-Current”)	**247**			
